# A new species of sea urchin associating clingfish of the genus *Dellichthys* from New Zealand (Teleostei, Gobiesocidae)

**DOI:** 10.3897/zookeys.740.22712

**Published:** 2018-02-27

**Authors:** Kevin W. Conway, Andrew L. Stewart, Adam P. Summers

**Affiliations:** 1 Department of Wildlife and Fisheries Sciences and Biodiversity Research and Teaching Collections, Texas A&M University, College Station, TX 77843, USA; 2 Museum of New Zealand Te Papa Tongarewa, 169 Tory Street, Wellington, New Zealand; 3 Friday Harbor Laboratories, University of Washington, Friday Harbor, Washington 98250, USA; 4 Burke Museum of Natural History and Culture, University of Washington, Seattle, Washington 98105, USA

**Keywords:** Taxonomy, marine fishes, Acanthomorpha, sexual dimorphism

## Abstract

A new species of clingfish, *Dellichthys trnskii* **sp. n.** is described on the basis of 27 specimens, 11.9–46.0 mm SL, collected from intertidal and shallow coastal waters of New Zealand. It is distinguished from its only congener, *D. morelandi* Briggs, 1955 by characters of the cephalic sensory system and oral jaws, snout shape, and colouration in life. A rediagnosis is provided for *D. morelandi*, which is shown to exhibit sexual dimorphism in snout shape.

## Introduction

The family Gobiesocidae contains over 160 species within 50 genera of predominately small-bodied marine fishes found in coastal areas of the Atlantic and Indo-Pacific Oceans (Briggs 1955; Conway et al. 2015), from the intertidal zone to ~500 meters (Hastings and Conway 2017). Seven species also are known to inhabit freshwater streams in the Neotropics (Briggs and Miller 1960; Conway et al. 2017a). Commonly referred to as clingfishes, members of this family generally exhibit a well-developed ventral adhesive disc (formed by elements of the paired-fin girdles (Guitel 1888)), with which they can attach to smooth or even heavily structured substrates with great tenacity (Wainwright et al. 2013; Ditsche et al. 2014).

Many species of clingfishes are reported to live in close association with echinoderms (Pfaff 1942; Teytaud 1971). This relationship may be obligate, as in the association between clingfishes of the genus *Discotrema* and certain crinoids (Briggs 1976; Fishelson 1966, 1974), or more facultative and dependent on life stage (Sakashita 1992; Goncçalves et al. 2002). For example, juveniles of the New Zealand urchin clingfish *Dellichthys morelandi* live directly underneath or in close proximity to the echinoid *Evechinus chloroticus* (Dix 1969, 1970). This association provides juveniles of *D. morelandi* with both protection from predators and also food, as confirmed by the presence of tube feet and/or pedicellaria in the stomachs of individuals dissected for diet analyses (Dix 1969; Russell 1983). Individuals of *D. morelandi* that have become too large to seek refuge beneath the spines of *E. chloroticus* instead occupy crevices between or under rocks or among shell debris (Paulin and Roberts 1992; Francis 2012).

Recent ichthyological surveys targeting clingfishes in intertidal and shallow sub-tidal areas along the coast of Northland (New Zealand) produced multiple individuals of *Dellichthys* from directly beneath or in close proximity to the sea urchin *E. chloroticus*. Subsequent investigation revealed that these specimens represent two species; one *D. morelandi* and the other an undescribed species, which is described herein.

## Materials and methods

Specimens used in this study were obtained from the following museum collections: Australian Museum, Sydney (**AMS**); Auckland War Memorial Museum, Auckland (**AIM**); Museum of New Zealand Te Papa Tongarewa, Wellington (**NMNZ**); and the Biodiversity Research and Teaching Collections, Texas A&M University, College Station (**TCWC**).

Head and body measurements reported follow Conway et al. (2014) and are expressed as percent of standard length (**SL**) or head length (**HL**). Adhesive disc papillae terminology follows Briggs (1955). Cephalic superficial neuromast row terminology follows Conway et al. (2017b) and cephalic lateral line pore terminology follows Shiogaki and Dotsu (1983), except that we also use numbers to refer to individual pores following Conway et al. (2017b).

Select specimens were cleared and double stained (**C&S**) for bone and cartilage investigation using the protocol of Taylor and Van Dyke (1985). Select specimens were reversibly stained using cyanine blue following Saruwatari et al. (1997) to aid examination of adhesive disc papillae. Specimens or parts thereof were observed and photographed using a ZEISS SteREO Discovery V20 stereomicroscope equipped with a ZEISS Axiocam MRc5 digital camera. Digital images taken with this set up were typically stacked using the ZEISS Axiovision software. Computed tomography (**CT**) scans of select specimens were also obtained at the Karel F. Liem BioImaging Center (Friday Harbor Laboratories, University of Washington) using a Bruker (Billerica, MA) SkyScan 1173 scanner with a 1 mm aluminum filter at 60 kV and 110 μA on a 2048 × 2048 pixel CCD at a resolution of 8.8 μm. Specimens were scanned simultaneously while inside a 50 ml plastic Falcon tube (Corning, NY), in which they were wrapped with cheesecloth moistened with ethanol (70 %) to prevent movement during scanning. The resulting CT data were visualized, segmented, and rendered in Horos (http://www.horosproject.org) and Amira (FEI). All digital images were processed using Adobe Photoshop and Adobe Illustrator.

Genomic DNA was extracted from muscle tissue or fin clips (stored in 95% ETOH) using a DNeasy Blood and Tissue Extraction Kit (Qiagen, Inc.) in accordance with the manufacturer’s protocols. A segment of the cytochrome c oxidase subunit I (COI) and the 12s ribosomal RNA (12S) was amplified using the primers LCO1490/ HC02198 (Folmer et al. 1994) and L1091/H1478 (Kocher et al. 1989), respectively. Parameters for PCR amplification followed Conway et al. (2017a). Genetic distances (uncorrected *p*-distances) were calculated based on COI and 12S sequences using PAUP v.4.0b10 (Swofford 2000).

## Systematics

### Dellichthys trnskii sp. n.

Taxon classificationAnimaliaGobiesociformesGobiesocidae

http://zoobank.org/1D5D5875-116E-4F15-9E65-322BE259F2DA

Figs 1 , 2A , 3 , 4A , 5A , 6 , 7A–B , 8A–C

#### Holotype.

**AIM MA73570**, 22.8 mm SL, New Zealand, Northland, Tutukaka, Pacific Bay, 35°37'07.2"S, 174°32'03.8"E, 0–2 meters depth, 8 March 2016, T. Trnski, I. Middleton, K.W. Conway, S. Hannam, & G. Short.

#### Paratypes.

All New Zealand. *Auckland*: **NMNZ P.028060**, 4 (1 CT [https://doi.org/10.17602/M2/M40584]), 20.1–29.7 mm SL; **NMNZ P.060626**, 2 (C&S), 21.0–25.0; Hauraki Gulf, Matatuahu Point, Tawharanui Peninsula, 0–5 meters depth (36°23'00.0"S, 174°49'00.0"E), 8 April 1992. *Bay of Plenty*: **NMNZ P.035572**, 1, 46.0 mm SL; Rurima Islets, 7–10 meters depth (37°49'47.0"S, 176°52'38.0"E), 02 June 1998. *Marlborough*: **NMNZ P.025671**, 2, 42.0–45.6 mm SL; **NMNZ P.060627**, 1 (C&S), 41.8 mm SL; Gorse Bay, Port Underwood (41°18'27.1"S, 174°09'39.7"E), 25 September 1989. *Hawke’s Bay*: **NMNZ P.057592**, 1, 32.7 mm SL; Bare Island (39°49'54.0"S, 177°01'30.0"E), 09 December 1991. – **NMNZ P.057600**, 1, 43.3 mm SL; south of Aramoana, 0–3 meters depth (40°09'42.0"S, 176°50'18.0"E), 19 January 1991. *Northland*: **AIM MA4341**, 1, 45.8 mm SL, Poraenui Point, Bay of Islands (35°11'34"S, 174°4'8"E), 15 December 1983. – **AIM MA6395**, 1, 11.9 mm SL, Kerikeri Inlet, Bay of Islands, 7 meters depth (35°12'0.0"S, 174°02'43.0"E), 28 Jan 1972. – **AIM MA7070**, 1, 33.0 mm SL, Te Puna off Mataka, Bay of Islands (35°09'0.0"S, 174°06'12.0"E), 20 March 1988. – **AIM MA73571**, 2 (ethanol preserved DNA vouchers), 17.0–20.0 mm SL; **TCWC 17264.03**, 1 (C&S), 18.0 mm SL, same as holotype. – **AIM MA75372**, 1, 18.8 mm SL, Rawhiti, Taupiri Bay (35°16'58.4"S, 174°17'38.0"E), 10 March 2016. – **AIM MA73573**, 1, 21.3 mm SL, Bland Bay, 0–3 meters depth (35°20'47.8"S, 174°21'57.6"E), 11 March 2016. – **NMNZ P.057601**, 1, 31.3 mm SL; north side of the Matapouri Peninsula, 0–8 meters depth (35°33'15.0"S, 174°30'00.0"E), 09 April 1992. – **TCWC 17171.04**, 1 (ethanol preserved DNA voucher), 25.5 mm SL, Tutukaka, rocky bay between Tutukaka reserve and Kukutauwhao Island (35°36'40.7"S, 174°32'29.8"E), 1 March 2015. *Wellington*: **NMNZ P.048189**, 1, 45.0 mm SL; Wellington Port, overseas passenger terminal (41°17'19.6"S, 174°47'09.5"E), 23 November 2001. – **NMNZ P.048197**, 1, 37.0 mm SL; Wellington Port, Burnham Wharf (41°18'42.0"S 174°48'12.0"E), 20 November 2001.

#### Other material.

**AMS I.34453-005**, 1, 20.0 mm SL; New Zealand: locality unknown.

#### Diagnosis.

*Dellichthys trnskii* is diagnosed by the following combination of characters: snout broad, short (length less than or equal to interorbital distance); upper and lower jaws equal in length or lower jaw only slight shorter than the upper; upper jaw teeth not visible or only few teeth visible in gap between upper and lower lip at tip of jaws when jaws are closed; patch of teeth on lingual surface of premaxilla roughly rectangular, with ~50 small conical teeth; skin fold on surface of snout directly posterior to fold of upper lip; postorbital lateral line canal pore 2 located directly above preopercular lateral line canal pore 3; tip of snout and lower jaw pale pink in life; dorsal and lateral surface of head light yellow to green in life; body pale orange to yellow in life; and median fins transparent and without faint brown reticulate markings in life.

#### Description.

**Figure 1.**
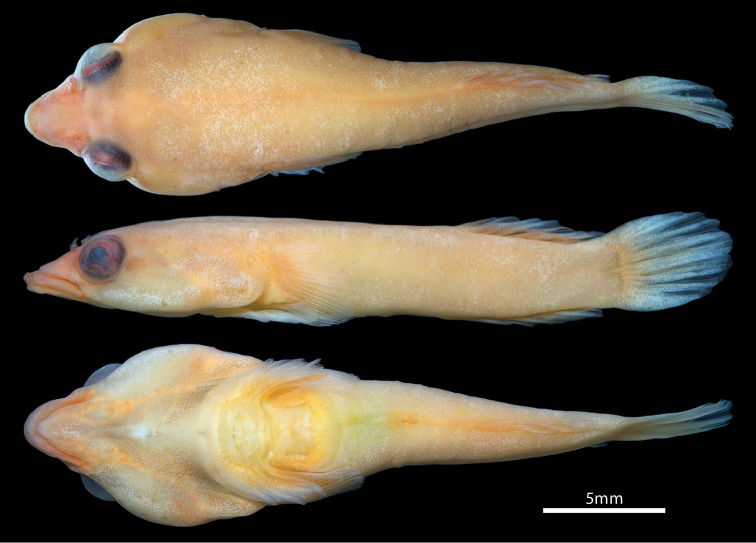
*Dellichthys trnskii*, AIM MA73570, holotype, male, 22.8 mm SL; New Zealand: Northland, Pacific Bay, Tutukaka Coast.

**Figure 2.**
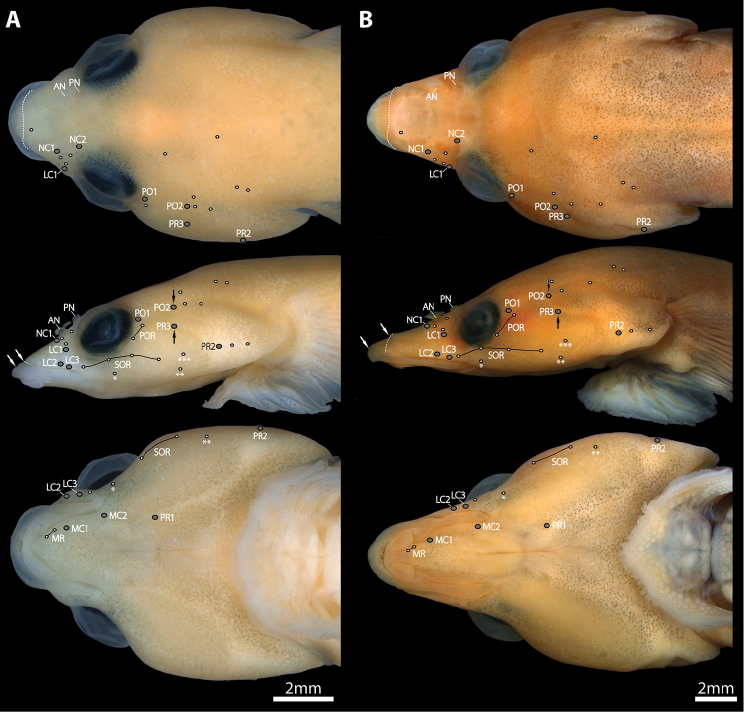
Head (in dorsal, lateral and ventral view) in members of *Dellichthys* highlighting position of cephalic lateral line canal pores (grey circles) and superficial neuromasts (white circles) on the left side of the head, and skin fold on snout. **A** *Dellichthys trnskii*, AIM MA73573, paratype, 21.3 mm SL **B** *Dellichthys morelandi*, TCWC 17264.02, 34.2 mm SL. Black arrows point to location of postorbital canal pore 2 (upper arrow) and preopercular canal pore 3 (lower arrow). White arrows point to location of posterior margin of upper lip (anterior arrow) and anterior margin of skin fold on snout (posterior arrow). White dashed line follows margin of skin fold on snout in dorsal and lateral view. Superficial neuromasts arranged in rows are connected by a thin black line. Superficial neuromasts on surface of body not highlighted. Abbreviations: AN, anterior nostril; LC1-3, lachrymal canal pores 1–3; MC1–3, mandibular canal pores 1–2; MG, mandibular row of superficial neuromasts; NC1–2, nasal canal pores 1–2; PN, posterior nostril; PO1–2, postorbital canal pores 1–2; POR, postorbital row of superficial neuromasts; PR1–3, preopercular canal pores 1–3; SOR, suborbital row of superficial neuromasts.

General body shape as in Figure 1. Morphometric characters listed in Table 1. Head large, slightly dorsoventrally compressed. Body moderately dorsoventrally compressed anteriorly, becoming increasingly laterally compressed posteriorly at region of dorsal and anal fins. Widest point of head wider than widest point of body (immediately behind head). Body width tapering gradually posteriorly. Eye large, positioned on dorsolateral surface of head; orbit visible in ventral view. Centre of eye closer to tip of snout than to posterior margin of operculum. Snout short, broad; anterior margin rounded. Transverse skin groove present across dorsal surface of snout; skin anterior to groove thin and transparent (Fig. 2A). Anterior nostril a small tubular opening, with short, thin blade-like flap extending from posterior margin. Posterior nostril tubular, situated along anterdorsal margin of orbit. Gill membranes united and free from isthmus.

**Table 1.** Select morphometric characters for *Dellichthys trnskii* (n=7) and *D. morelandi* (n=12).

	*Dellichthys trnskii*				*Dellichthys morelandi*		
	Holotype	Range	Mean	St. Dev.	Range	Mean	St. Dev.
Standard Length (SL)	22.8	20.1–31.7			31.0–65.5		
**In % of SL**							
Head length (HL)	41.6	40.4–45.5	42.1	1.8	38.3–43.6	41	1.6
Body depth	15.3	14.2–16.4	15.2	0.8	12.3–15.5	13.7	1
Predorsal length	72.8	72.8–80.1	75.4	2.6	74.3–79.2	76.3	1.6
Preanal length	75.4	73.7–79.1	78.5	1.8	73.8–80.5	76.7	1.9
Preanus length	62.3	62.2–66.3	63.6	1.8	62.2–71.2	66.1	2.6
Anus to disc	8.8	8.8–12.6	10.4	1.3	11.2–15.2	13.1	1.3
Anus to anal fin	17.5	17.3–24.5	19.2	2.7	10.5–15.2	12.8	1.8
Caudal peduncle length	10.1	7.7–10.1	9.1	0.8	7.2–9.4	8.2	0.7
Caudal peduncle depth	10.1	9.7–11.8	10.5	0.7	8.4–10.3	9.4	0.5
Disc length	24.1	22.8–27.8	25.9	1.9	22.6–25.9	24.3	1
Disc width	24.5	22.0–25.9	23.8	1.3	20.4–23.5	21.9	1.1
**In % of HL**							
Head depth at orbit	31.6	25.2–31.6	28.5	2.1	21.3–24.6	23.4	1
Head width at orbit	45.3	36.1–45.3	40.6	3	32.5–39.3	35.3	2.3
Head width at widest point	68.4	58.5–68.4	62.7	4.2	51.1–65.3	55.9	4.3
Interorbital width	26.3	20.5–27.7	23.9	3.3	17.9–23.2	19.9	2
Snout length	28.4	23.9–28.8	26.7	1.8	25.6–33.7	31.2	2.3
Eye diameter	22.1	18.8–23.5	20.9	1.7	13.9–19.8	16.3	1.8

**Figure 3.**
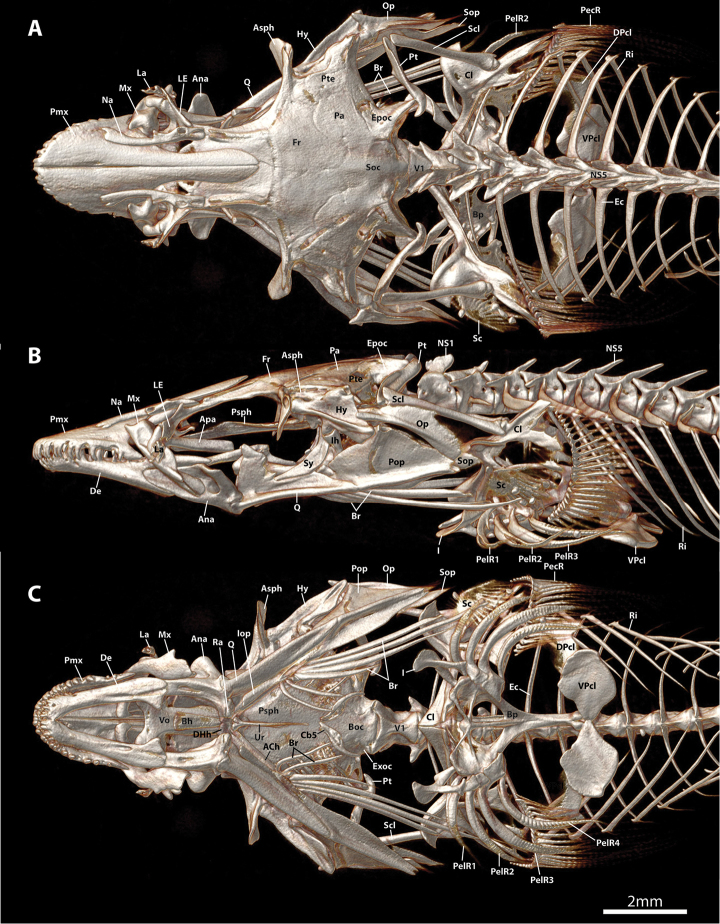
CT scanned anterior skeleton, including cranium, paired-fin girdles and abdominal region of vertebral column, of *Dellichthys trnskii*, NMNZ 028060, paratype, 25.0 mm SL. **A** Dorsal view **B** Lateral view **C** Ventral view. Single scale bar shared by **A–C**. Abbreviations: ACh, anterior ceratohyal; Ana, anguloarticular; Apa, autopalatine; Asph, autosphenotic; Bh, basihyal; Boc, basioccipital; Bp, basipterygium; Br, branchiostegal rays; Cb5, ceratobranchial five; Cl, cleithrum; De, dentary; DPcl, dorsal postcleithrum; Ec, epicentral; Epoc, epioccipital; Exoc, exoccipital; Fr, frontal; Hy, hyomandibular; I, spinous pelvic-fin ray; Ihc, interhyal; Iop, interopercle; La, lachrymal; LE, lateral ethmoid; Mx, maxilla; Na, nasal; NS, neural spine; Op, opercle; Pa, parietal; PecR, pectoral-fin ray; PelR, pelvic-fin ray; Pmx, premaxilla; Pop, preopercle; Psph, parasphenoid; Pt, posttemporal; Pte, pterotic; Q, quadrate; Ra, retroarticular; Ri, rib; Sc, scale; Scl, supracleithrum; Soc, supraoccipital; Sop, subopercle; Sy, symplectic; Ur, urohyal; V, vertebral centrum; VPcl, ventral postcleithrum.

**Figure 4.**
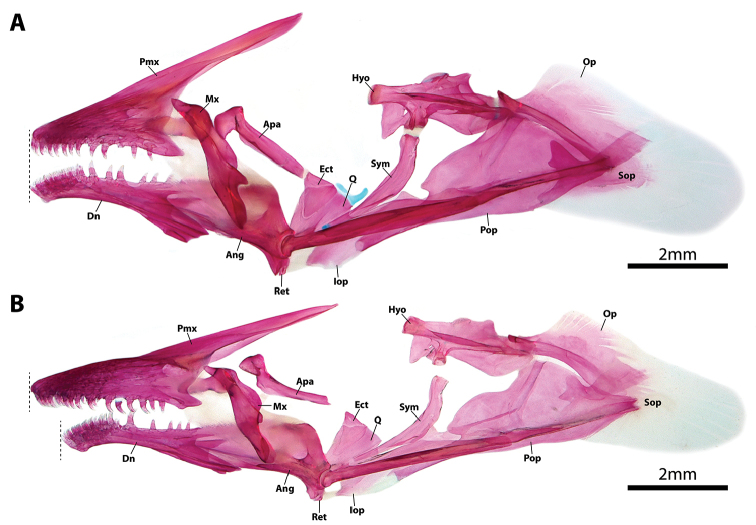
Hyopalatine arch and opercular series (right side in lateral view [image reversed]) in members of *Dellichthys*. **A** *D. trnskii*, NMNZ P.062699, paratype, 41.8 mm SL **B** *D. morelandi*, NMNZ P.018388, 39.0 mm SL. Dashed lines indicate anterior extent of upper and lower jaws. Abbreviations: Ang, anguloarticular; Apa, autopalatine; Dn, dentary; Ect, ectopterygoid; Hyo, hyomandibular; Iop, interopercle; Mx, maxilla; Op, opercle; Pmx, premaxilla; Pop, preopercle; Q, quadrate; Ret, retroarticular; Sop, subopercle; Sym, symplectic.

**Figure 5.**
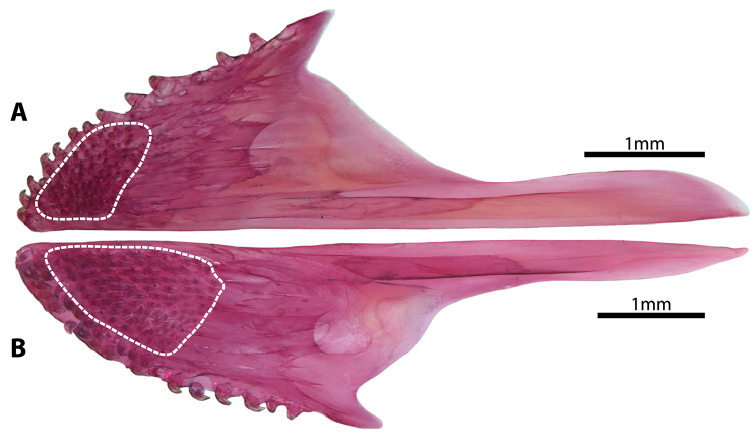
Premaxilla (right side in ventral view [image reversed]) in members of *Dellichthys*. **A** *D. trnskii*, NMNZ P.062699, paratype, 41.8 mm SL **B** *D. morelandi*, NMNZ P.018388, 39.0 mm SL.

Mouth terminal, small; posterior tip of upper jaw reaching imaginary vertical line through anterior margin of orbit when mouth closed. Upper lip narrow; thickest along lateral margin of upper jaw; thinnest at snout tip. Lower lip thin at jaw symphysis; expanded into fleshy lobes adjacent to symphysis. Premaxilla with outer row of larger conical teeth with strongly recurved tips (Figs 3B, 4A, 5A) and medial, roughly rectangular patch of ~50 smaller conical teeth on lingual surface posterior to outer row of larger teeth (Fig. 5A). Dentary with broad patch of conical teeth with recurved tips anteriorly, tapering to single row of larger conical teeth posteriorly (Fig. 4A). Pharyngeal jaws comprising patch of 16–18 small conical teeth with slightly recurved tips on pharyngobranchial toothplate 3 and two rows of 5–8 small conical teeth with slightly recurved tips along ceratobranchial 5. 10–12 gill rakers located along anterior and posterior edge of ceratobranchials 2–3 and anterior edge of ceratobranchial 4; 7 gill rakers located along anterior edge of ceratobranchial 1. Gill filaments associated with ceratobranchials 1–4 (3.5 gill filaments of Briggs (1955)); ceratobranchial 1–3 each with holobranch; hemibranch only on ceratobranchial 4. Basihyal elongate, widest anteriorly (Fig. 3C); anterior edge tipped with cartilage. Branchiostegal rays 6; two anteriormost rays articulating medially with hyoid bar along anterior ceratohyal; posterior rays articulating with hyoid bar laterally, including 3 along posteriormost part of anterior ceratohyal and 1 straddling junction between anterior and posterior ceratohyals (Fig. 3C). Anteriormost branchiostegal rays shorter than posterior rays; orientated with posterior tips directed towards ventral midline. Two posteriormost branchiostegal rays approximately twice as long as short anterior rays; orientated with posterior tips directed towards posterior. Intervening rays intermediate in length; orientated with posterior tips directed towards posterior.

Cephalic lateral-line system with 2 pores in nasal canal; 2 pores in postorbital canal; 3 pores in lachrymal canal; 3 pores in preopercular canal; 2 pores in mandibular canal (Fig. 2A). Postorbital canal pore 2 located directly above preopercular canal pore 3. Mandibular and preopercular canals continuous; connected via unossified canal; anteriormost pore of preopercular canal (PR1) located at center of unossified canal between anguloarticular and preopercle (Fig. 2A). Superficial neuromasts on head isolated or arranged in rows (Fig. 2A). 4 superficial neuromasts in suborbital row; 2 superficial neuromasts in postorbital row; 2 superficial neuromasts in mandibular row.

**Figure 6.**
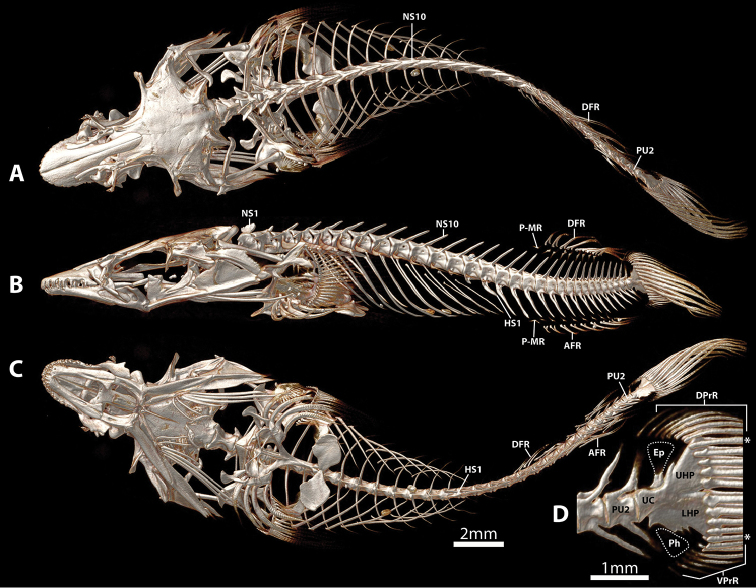
CT scanned skeleton of *Dellichthys trnskii*, NMNZ P.028060, paratype, 25.0 mm SL. **A** Dorsal view **B** Lateral view **C** Ventral view **D** Caudal fin skeleton, lateral view. Asterisks indicate position or upper- and lowermost principal caudal-fin rays in D. Outline of poorly ossified portion of epural and entire parhypural indicated by white dotted line in D. Single scale bar shared between A-C. Abbreviations. AFR, anal-fin ray; DFR, dorsal-fin ray; DPrR, dorsal procurrent caudal-fin ray; Ep, epural; HS1, first hemal spine (14^th^ vertebral centrum); LHP, lower hypural plate; NS, neural spine, number indicates associated vertebral centrum; P-MR, proximal-middle radial; PrR PU2, second preural centrum; UC, ural centrum; UHP, upper hypural plate; VPrR, ventral procurrent caudal-fin ray.

Dorsal-fin rays 9. Anal-fin rays 7 (1), 8 (2) or 9 (1). Principal caudal-fin rays 5+5, dorsal procurrent rays 6 (2) or 7 (2), ventral procurrent rays 5 (1), 6 (2) or 7 (1). Pectoral-fin rays 22 (3) or 23 (1). Pelvic-fin rays I,4. All fin rays unbranched and segmented. Caudal fin rounded, tips of principal caudal fin rays extended slightly beyond fin margin. Caudal-fin skeleton comprised of upper and lower hypural plates; epural and parhypural poorly ossified, triangular and similar in size (Fig. 6D). Dorsal-fin origin opposite anal-fin origin. First dorsal-fin pterygiophore inserted between neural spines of vertebrae 15/16. First anal-fin pterygiophore inserted between hemal spines of vertebrae 15/16. Total number of vertebrae 30 (3) or 31 (1), consisting of 13 abdominal and 18 (3) or 19 (1) caudal vertebrae (Fig. 6). Ribs 10, associated with vertebrae 3–12. Epicentrals 17 (3), associated with vertebrae 2–18, or 21, associated with vertebrae 2–22.

**Figure 7.**
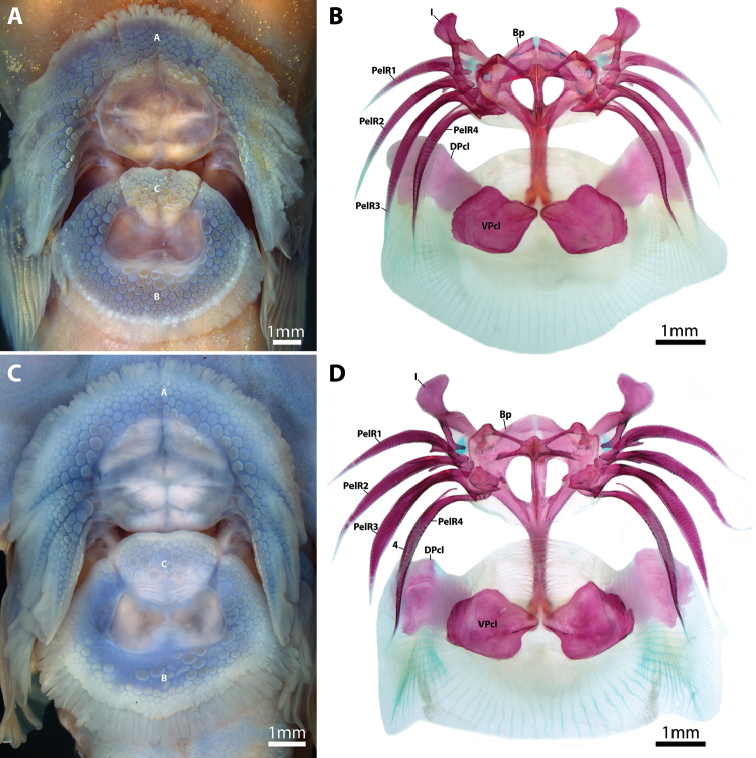
Surface features (**A, C**) and internal supporting skeleton (**B, D**) of the adhesive disc in species of *Dellichthys* in ventral view (anterior to top of page). **A** *D. trnskii*, AIM MA4341, 45.8 mm SL **B** *D. trnskii*, NMNZ P.062699, paratype, 41.8 mm SL **C** *D. morelandi*, TCWC 17269.03, 37.1 mm SL **D** *D. morelandi*, NMNZ P.018388, 39.0 mm SL. Abbreviations: A, disc region A; B, disc region B; Bp, basipterygium; C, disc region C; DPcL, dorsal postcleithrum; I, pelvic-fin spine; PelR1–4, pelvic-fin rays 1–4; VPcL, ventral postcleithrum.

Adhesive disc large, double (Fig. 7A); anterior and posterior margin weakly crenulated. Disc region A with 5–6 transverse rows of papillae. Disc region B with 6–7 transverse rows of papillae. Disc region C with 4–5 rows of papillae. Papillae of disc region A decreasing in diameter towards outer margin of disc. Papillae of disc region B and C decreasing in diameter towards outer margin of inner disc. Dorsal postcleithrum a thin irregular shaped bone; larger than ventral postcleithrum (Fig. 7B). Ventral postcleithrum irregular in shape; lateral edge rounded; medial edge roughly triangular, with point directed toward ventral midline (Fig. 7B). Fimbrae along posteroventral margin of dorsal postcleithrum and posterior margin of ventral postcleithrum well-developed. Skin associated with last pelvic-fin ray attaching to base of pectoral fin opposite 5th lowermost pectoral-fin ray. Skin over base of ventral pectoral-fin rays smooth.

#### Colouration.

In alcohol, body background colour pale yellow. Median fins pale yellow to white along bases, transitioning to hyaline along distal margins. Paired fins hyaline; papillae on adhesive disc translucent white. In formalin and shortly after initial transfer to alcohol (Fig. 1), body background colour pale orange to yellow with darker orange markings along dorsal midline and ventral midline posterior to adhesive disc. Snout and lips orange. Orange to light brown stripe on lateral side of head posterior to orbit. Dorsal and anal fins orange along base, transitioning to white along distal margins. Base and center of caudal fin pale orange, transitioning to white along distal margins. Pectoral fin hyaline. Distal margin of pelvic fin whitish; papillae on adhesive disc light orange.

**Figure 8.**
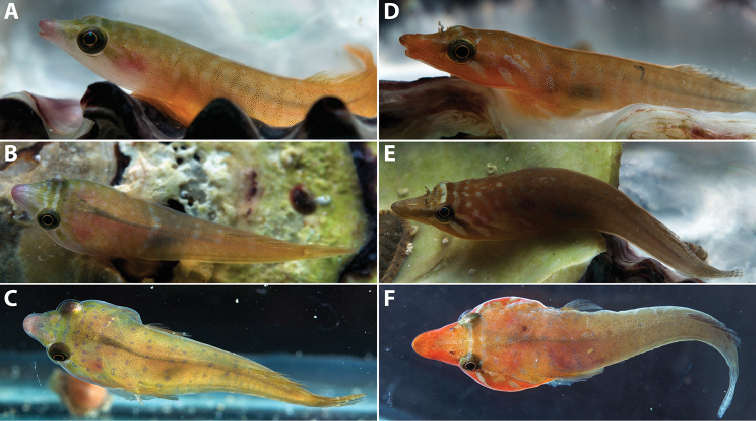
*Dellichthys trnskii* (**A–C**) and *D. morelandi* (**D–F**) photographed in an aquarium soon after collection. **A–B** AIM MA73570, holotype, male, 22.8 mm SL; Pacific Bay, Tutukaka Coast **C** AIM MA73571, paratype, female, 20.0 mm SL; Pacific Bay, Tutukaka Coast **D** TCWC 17264.02, male, 33.8 mm SL; Pacific Bay, Tutukaka Coast **E** TCWC 17264.02, potential female, 29.0 mm SL; Pacific Bay, Tutukaka Coast **F** TCWC 17269.03, male, 37.1 mm SL; Rawhiti, Taupiri Bay.

In life, background colour translucent orange to pale yellow (Fig. 8A–C). Lateral body surface with faint to distinct irregular white to pale blue lines that may or may not connect with counterparts along dorsal midline. In some individuals (potentially female), irregular lines replaced by irregular rows of small white to pale blue spots (Fig. 8C). Light brown pigment surrounding nerve cord and darker content in stomach visible through body. Dorsal surface of head translucent light yellow to pale green with three or four white to pale blue lines that become more obvious anteriorly. Lateral surface of head posterior to orbit with two white to pale blue lines flanking a central light brown to pale green region (equivalent to orange to light brown stripe on lateral side of head posterior to orbit described above for specimens in formalin). In some individuals (potentially female), white to pale blue lines on dorsal and lateral surfaces of head are replaced by rows of small white to pale blue spots. Tip of snout and adjacent portions of lips pink. Iris orange. Fins clear to translucent orange/yellow.

#### Distribution.

**Figure 9.**
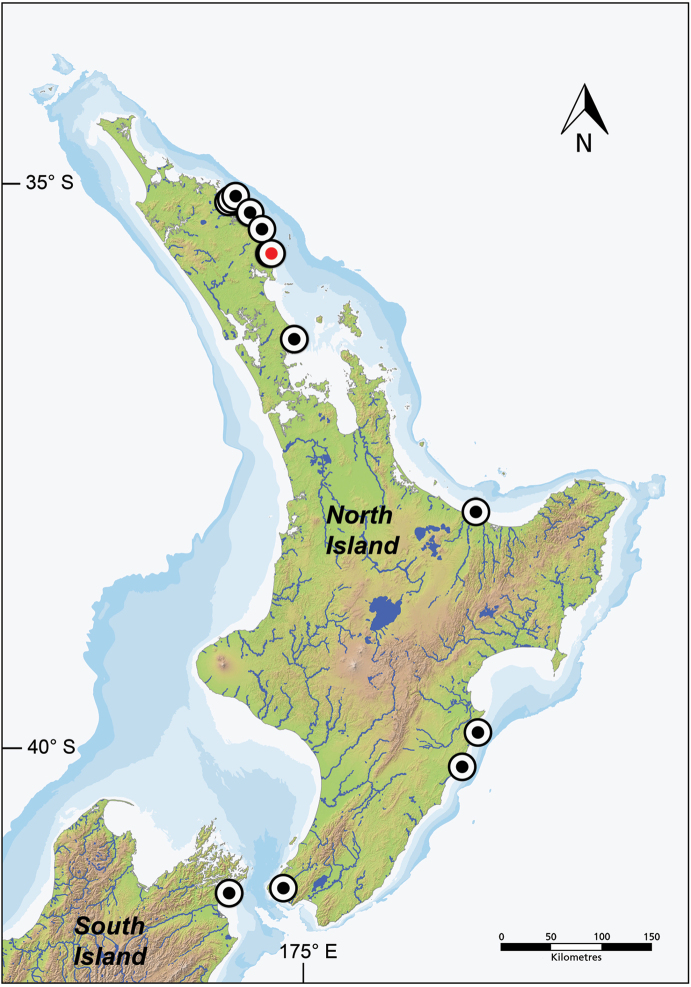
Distribution of *Dellichthys trnskii*. Type locality marked with a red dot.

*Dellichthys trnskii* is endemic to New Zealand coastal waters, currently known only from shallow (0–7 meters in depth) waters along the northeastern coast of both the North Island (Auckland, Bay of Plenty, Hawke’s Bay, Northland, and Wellington) and South Island (Marlborough Sounds) (Fig. 9). Its occurrence further south may be confirmed by further sampling and by a better understanding of the differences between the two species.

#### Notes on biology

. At the type locality, *D. trnskii* was found primarily under rocks or boulders covered with filamentous algae or low macroalgae often in close proximity to the sea urchin *Evechinus chloroticus*. Small dense objects, possibly sand grains, are visible in the pharyngeal cavity and gut of the CT scanned paratype (NMNZ P.028060, 25.0 mm SL; Figs 3, 6). A single ctenoid scale also is lodged in the opercular opening of this individual (Fig. 3). Whether this scale was ingested or entered the opercular opening subsequent to capture is difficult to confirm. The specimen was collected with a large number of associated sub-tidal species including triplefins, some of which could have shed scales in the bag.

#### Sexual dimorphism.

No obvious sexual dimorphism is present in the available material. Potential sexual dichromatism is described above in the section on colouration.

#### Etymology.

Named for Tom Trnski, who played a key role in the discovery of the new species by collecting in depths beyond the reach of the first author. A noun in the genitive.

#### Genetic Distances.

The sequences of COI (684bp) obtained from two specimens of *D. trnskii* (Genbank numbers [GB#] MF621939-40) were identical and differed from sequences obtained from six specimens of *D. morelandi* (GB# MF621941-44, MF318544-45) by 11.7 % (uncorrected *p*-distance). Similarly, the sequences of 12S (365bp) obtained from three specimens of *D. trnskii* (GB# MF621933-35) were identical and differed from sequences obtained from five specimens of *D. morelandi* (GB# MF318559-60, MF621936-38) by 3.4% (uncorrected *p*-distance).

#### Comparisons.

*Dellichthys trnskii* is most easily distinguished from *D. morelandi* by features of the colour pattern in life (Fig. 8), including a pale orange to yellow background colour on the body (vs. light brown to dark orange, red or purplish), areas between white to pale blue markings (most commonly stripes) on dorsal and lateral surface of head light yellow to green (vs. brown to dark orange or red), tip of snout and lower jaw pale pink (vs. brown to dark orange or red), and the absence (vs. presence) of faint brown reticulate markings on the median fins.

*Dellichthys trnskii* is further distinguished from *D. morelandi* by features of the oral jaws, including having the upper and lower jaws equal in length or the lower jaw only slight shorter than the upper, with few upper jaw teeth visible in the gap between the upper and lower lip when the jaws are closed (vs. upper jaw notably longer than lower jaw, with many upper jaw teeth visible in the gap between the upper and lower lip when the jaws are closed) (Fig. 4), a small, roughly rectangular patch of ~50 small conical teeth on the lingual surface of the premaxilla that flanks the posterior margin to the larger conical teeth along the outer margin of the bone (vs. large, roughly triangular patch of ~90 small conical teeth that extends over much of the anterolingual surface of the premaxilla) (Fig. 5). *Dellichthys trnskii* also can be distinguished from *D. morelandi* by its slightly shorter snout (snout length 24–29 % HL vs. 26–34 % HL in *D. morelandi*), the length of which is equal to or less than the interorbital distance (vs. snout length greater than interorbital distance), and by having the transverse skin fold on the surface of snout located directly posterior to the fold of the upper lip (vs. transverse skin fold on the surface of the snout separated from the fold of the upper lip by a broad band of thin, transparent skin). Finally, *D. trnskii* is distinguished from *D. morelandi* by the location of postorbital lateral line canal pore 2, which is located directly above preopercular lateral line canal pore 3 (vs. postorbital lateral line canal pore 2 anterior to preopercular lateral line canal pore 3) (Fig. 2).

#### Remarks.

Briggs (1955) erected *Dellichthys* for the sole inclusion of *D. morelandi*, which he considered to be an “interesting species with no known close relatives” (Briggs 1955: 15). We assign the new species to *Dellichthys* because it exhibits all of the diagnostic characters listed by Briggs (1955:14), including: a narrow upper lip, separated by a broad frenum at tip of snout; small, sharp, conical teeth arranged in a deep patch on both the upper and lower jaw, tapering to a single row of larger, strongly recurved, “canine-like” teeth posteriorly; a relatively high number of narrow pointed gill-rakers on the second gill arch (10–12 in *D. trnskii*; 14 listed by Briggs for *D. morelandi*, 1955); a poorly developed fleshy pad at lowest part of pectoral-fin base; and ventral postcleithrum with a “characteristic” shape in ventral view (i.e., lateral margin rounded and medial margin roughly triangular; Briggs 1955: fig. 44).

Briggs (1955: 14) also listed the absence of the subopercle as diagnostic for *Dellichthys* and considered the opercle to form the terminal element of the operculum in *D. morelandi*. Our investigation of the osteology of *Dellichthys morelandi* and *D. trnskii* has revealed the subopercle to be present (Figs 3A,B, 4). In both cases, the subopercle is comprised of a small, heavily ossified anterior part at the point of articulation with the opercle and preopercle, and a very poorly ossified posterior portion, represented by a thin, yet extensive, lamina of dermal bone that does not take up alizarin red S when cleared and double stained (Fig. 4) nor render well in reconstructions of the CT scan data (e.g., Fig. 3). The poorly ossified posterior margin to the subopercle in *D. morelandi* and *D. trnskii* differs markedly from the heavily ossified and often spine-like posterior margin to the subopercle present in other gobiesocids (e.g., see fig. 12 in Conway et al. (2017c)) and we consider this unique condition (not absence) of the subopercle to be diagnostic for *Dellichthys*.

Though Briggs (1955) provided a detailed diagnosis for *Dellichthys*, derived from multiple external and internal morphological features, the diagnosis provided for *D. morelandi* (also on pg. 14) is relatively short and lists characters that apply to both *D. morelandi* and *D. trnskii*. A rediagnosis for *D. morelandi* is provided below.

*Dellichthys trnskii* is sympatric with *D. morelandi*, at least along the coast of Northland, and specimens of the two species were commonly collected from within close proximity, in some cases from under the same rock. Paulin and Roberts (1992: 52) described the head and body colour of *D. morelandi* as “purple or cream with blue spots and a band of pale colour across the nape”. Stewart (2015: 1545) used the same description for juveniles of *D. morelandi* but described adults as “more olive grey-brown, sometimes flushed with red to orange around ventral part of head.” Given that *D. morelandi* and *D. trnskii* occur together and are similar in appearance, we suspect that these previous published descriptions of live colouration in *D. morelandi* are based on observations of both species, with purple specimens representing *D. morelandi* (e.g., see Francis 2012: 54) and cream specimens representing *D. trnskii* (e.g., see Fig. 8C). We note here that the cream coloured specimen figured in the account for *D. morelandi* in Stewart (2015: 1545, fig. 218.2) is instead a small specimen of *Trachelochismus melobesia*.

### Dellichthys morelandi

Taxon classificationAnimaliaGobiesociformesGobiesocidae

Briggs, 1955

#### Material examined.

All New Zealand. *Gisborne*: **NMNZ P.001574**, holotype, 35.8 mm SL; Lottin Point. *Auckland*: **AIM MA995**, 1, 38.8 mm SL; Waitemata Harbour, Torbay Reef (36°41'42.0"S, 174°45'42.0"E), 13 May 1968. – **AIM MA5414**, 5, 28.0–36.2 mm SL; Waitemata Harbour, Okoromai Bay, Whangaparoa Peninsula, 22 January 1985. – **AIM MA27860**, 1 (DNA voucher), 18.7 mm SL; Waterfall Bay, Manukau Harbour (37°01'44.4"S, 174°32'17.9"E), 9 April 2008. *Chatham Islands*: **AIM MA 93927**, 1, 45.4 mm SL; Waitangi Ellice Point (43°56'36.0"S, 176°33'54.0"W), 28 March 1995. *Northland*: **AIM MA5198**, 2, 35.2–36.0 mm SL; Whangaruru, Bland Bay (35°20'24.0"S, 174°22'24.0"E), 22 February 1984. – **AIM MA5331**, 3, 27.5–42.7 mm SL; Bay of Islands, Urapukapuka Island, Albert Passage (35°13'18.0"S, 174°14'36.0"E), 22 August 1984. – **AIM MA5345**, 2, 31.5–32.7 mm SL; Cape Wiwiki, Bay of Islands (35°09'36.0"S, 174°07'36.0"E), 23 August 1984. – **AIM MA7024**, 5, 22.8–60.5 mm SL; Te Puna off Mataka, Bay of Islands (35°09'0.0"S, 174°06'12.0"E), 20 March 1988. – **AIM MA77665**, 1, 51.6 mm SL; Deep Water Cove, Bay of Islands (35°11'42.0"S, 174°18'0.0"E), 8 September 1992. – **AIM MA656096**, 1, 27.4 mm SL; Three Kings Islands, Great Island (34°09'08.9"S, 172°07'50.5"E), 18 April 2013. – **NMNZ P.018388**, 15 (2 C&S), 29.0–46.4 mm SL; Motukokako Island (35°09'00.0"S, 174°20'00.0"E), 8 February 1986. – **TCWC 17173.03**, 1, 21.0 mm SL; Matapouri, Mermaid Pool (35°33'32.1"S, 174°30'51.3"E), 2 March 2015. – **TCWC 17174.03**, 1, 23.7 mm SL; Tutukaka, Dolphin Bay (35°37'33.4"S, 174°32'33.4"E), 2 March 2015. – TCWC 17264.02, 3, 20.0–33.8 mm SL; Tutukaka, Pacific Bay (35°37'07.2"S, 174°32'03.8"E), 8 March 2016. –**TCWC 17264.02**, 3, 21.0–35.0 mm SL. –**TCWC 17269.03**, 1, 37.1 mm SL; Rawhiti, Taupiri Bay (35°16'58.4"S, 174°17'38.0"E), 10 March 2016. – **TCWC 17615.04**, 3, 18.2–32.0 mm SL; **TCWC 17615.14**, 2 (C&S), 18.5–22.0 mm SL; Tutukaka, rocky bay between Tutukaka reserve and Kukutauwhao Island (35°36'40.7"S, 174°32'29.8"E), 11 March 2016. *Southland*: **AIM MA6548**, 1, 42.8 mm SL; Chalky Sound (46°03'0.0"S, 166°31'0.0"E), 23 May 1986. Wellington: **NMNZ P.030622**, 4, 28.1–65.5; Castlepoint, Wairarapa (40°54'00.0"S, 176°14'00.0"E), 14 December 1992. – **NMNZ P.030626**, 5 (2 CT, 1 male [https://doi.org/10.17602/M2/M37807], 1 female [https://doi.org/10.17602/M2/M37808]), 41.4–61.4; same as NMNZ P.030622, 11 November 1992.

#### Diagnosis.

*Dellichthys morelandi* is diagnosed by the following combination of characters: snout spatulate, long (length greater than interorbital distance); lower jaw shorter than upper jaw; teeth at tip of upper jaw visible in gap between upper and lower lip at tip of jaws when jaws are closed; patch of teeth on lingual surface of premaxilla roughly triangular, with ~90 small conical teeth; skin fold on surface of snout located at approximately one quarter of the distance from snout tip to anterior margin of eye, widely separate from fold of upper lip by a broad band of thin, transparent skin; postorbital lateral line canal pore 2 located anterior to imaginary horizontal line through preopercular lateral line canal pore 3; dorsal and lateral surface of head light brown to bright orange or red in life; body light brown to dark orange, red or purplish in life; faint brown reticulate markings on median fins in life.

#### Remarks.

Briggs (1955) described *D. morelandi* based on 14 specimens all from Lottin Point (East Cape). Though we have examined more specimens than were available to him, the morphometric and meristic characters reported herein (Table 1) are consistent with those in the original description.

**Figure 10.**
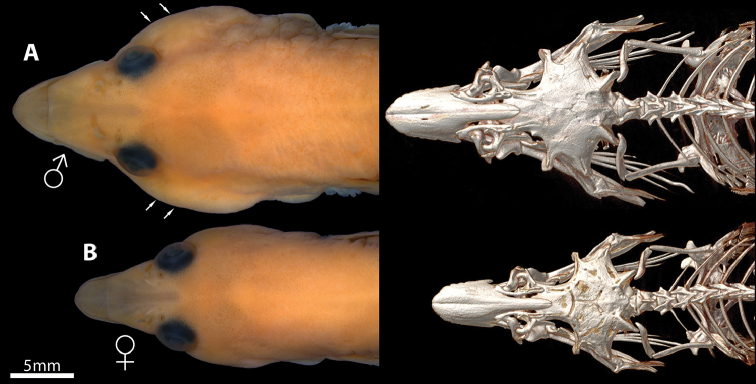
Alcohol preserved specimen (left) and corresponding CT (right) of a male and female of *Dellichthys morelandi*, NMNZ P.030626 **A** male, 60.0 mm SL **B** female, 42.0 mm SL. White arrows point to expanded cheek region of male in A.

Briggs (1955:14) made several observations on the snout of *D. morelandi*, which he described as “protruding, spatulate” and “distinctive”. Briggs (1955) made no mention of sexual dimorphism in relation to the snout of *D. morelandi* but based on the material that we have examined there is a clear difference in snout shape between the sexes, with males possessing wider snouts than females when viewed from above (Fig. 10). In addition to a wider snout, males also exhibit a wider head than females, which may be related to an increase in the size of the muscles of the adductor mandibulae complex. These differences in snout and head profile between the sexes do not appear to be mirrored in the cephalic skeleton, which is similar in males and females (Fig. 10). Sexual dimorphism in head and snout shape has been reported for a number of gobiesocids (e.g., Guitel 1888; Pfaff 1942; Sakashita 1992), is likely related to male nest guarding and parental care (e.g., Coleman 1998; Pires and Gibran 2011), and is probably more widespread than known currently within this fascinating group of fishes.

## Supplementary Material

XML Treatment for Dellichthys trnskii

XML Treatment for Dellichthys morelandi
